# Alumni Reunion

**Published:** 1899-03

**Authors:** 


Monthly Review of Medicine and Surgery.

*EDITORS:*

THOMAS LOTHROP, M. D. - WM. WARREN POTTER, M. D.

All communications, whether of a literary or business nature, should be addressed to the managing editor: 284 Franklin Street, Buffalo, N. Y.

Vol. XXXVIII. MARCH, 1899. No. 8.

### ALUMNI REUNION.

SOMETIME ago the officers in charge of the Alumni Association of the Medical Department of the University of Buffalo conceived the idea of holding a mid-winter reunion. This conception was carried to a most successful conclusion on Wednesday evening, February 22, 1899.

The selection of this patriotic day for the gathering of the sons and daughters of the college was most appropriate, for there is no more fit way to observe the occasion than for scientific or collegiate societies to come together in commemoration of the birthday of the "Father of his Country."

The executive committee, Dr. A. T. Lytle, chairman, and Drs. G. W. Wende, H. U. Williams, Charles Cary, Eli H. Long and A. T. Kerr, associates, deserve great credit for the admirable manner in which the ceremonies were conducted. The music was good, the supper served in the library was excellent in material and service, and the literary part of the entertainment proved most satisfactory.

At the appointed hour, 8.30 o'clock, the guests began to arrive and soon the corridors and rooms were filled with an enthusiastic throng of men and women, who were greeting each other once more under the roof of their alma mater. After an exchange of greetings, they were conducted through the various laboratories, where interesting details were observed, each teacher in charge being at his post to point out or explain some of the newer methods of instruction now in vogue. The State Laboratory, with Dr. H. R. Gaylord in immediate charge, where investigations relating to the origin and propagation of cancer are going on, proved of special interest to many. This was established last year under the supervision of Dr.

Roswell Park and most excellent progress is making in the fulfilment of its object. The chemic, microscopic, anatomic, physiologic and pathologic laboratories were all thrown open and it was the consensus of opinion among the visitors that nothing more complete than these can be found in any medical institution in this country.

In Alumni Hall, Prof. H. M. Hill exhibited stereopticon views of some of the work that was under study and investigation in these laboratories. This attracted attention up to the hour of 9.30 o'clock, which had been appointed for the beginning of the literary exercises. At the stroke of the gong, Prof. M. D. Mann, dean of the faculty, took the chair and delivered the following address :

In behalf of the medical faculty, I extend to you a hearty greeting. You have been asked here tonight because your alma mater wishes to show to her sons and daughters that she still takes a deep interest in them, and because she wishes to excite a warmer and more cordial feeling in her offspring toward herself. The members of a family who rarely get together, who see little of each other, or of their old home, cannot be expected to have much mutual interest or deep family feeling.

The alumni of a medical college are somewhat differently placed from the graduates of other colleges. It, unfortunately, too often happens, that in this country medical schools are entirely dependent for their support on the income derived from students' fees. With out stopping to think of the cost of properly conducting such schools, students, finding that the faculty insist on the payment of rather large fees, are apt, during their days of pupilage, to look upon the faculty as a business corporation, gotten up, more or less, for the purpose of getting their money. This engenders, in a way, a feeling of antagonism, which is still further heightened by the professional rivalry or competition, which they soon find to exist to a greater or less degree between themselves and the faculty when they become practitioners. Thus the alumni are apt to think of the managing faculty both as rivals and as members of a combine, managing the college solely for their own advantage and in their own interests—in other words, as a purely selfish, money-making institution. It is very natural that, feeling in this way, interest should flag or be wanting, and that in time feelings of hostility and antagonism to the college and its management should grow up amongst those who, by nature and inheritance, should be its best friends.

It is not very long since the absurd idea was prevalent that this college was owned by one man and run solely in his interests. It may not be out of place to remind you that the permanent faculty are appointed by the Council of the University, who own all the stock, are subject to them and hold office at their pleasure, and that to each of them is given an equal power and equal interests.

Now let us see if the sentiments attributed to some of the alumni are really well founded.

Modern education has made great demands on our educational institutions; we hear constant appeals from our great universities for more money, more buildings, more apparatus and more and higher-priced men. As a result, great efforts are being put forth and large endowments are being accumulated by the old institutions, and new ones are being founded, with great wealth already in their treasuries. Only recently the needs of medical education have begun to be appreciated. Already some of the old medical schools have been heavily endowed, or have been absorbed into the great universities, where large sums are appropriated for their maintenance. This has been especially the case in our own state. In this way we come to have the richly supported medical departments of Columbia, Cornell, Harvard and Johns Hopkins Universities. But, while this has happened in a few instances, the majority of the old schools are still forced to go on in the same way and, notwithstanding their entirely inadequate foundations, to meet the rivalry of the rich schools and the demands of modern education.

This is our position. To be sure, owing to the generosity of our citizens, we have a fine building and a good equipment. But this means greatly increased expenses in every way. The cost of maintaining such a building is vastly greater. Modern teaching demands laboratory work. To run laboratories costs much money; and, what is more, it needs men who will devote their entire time to the work, and must, therefore, get salaries which will properly support them. In endeavoring to follow out and fulfil these requirements, your faculty have done all in their power. The salary list now paid to teachers and employees, outside the permanent faculty, amounts to nearly $7,000 and yet some of the salaries are entirely inadequate, and should be increased if we desire to keep the men. The improvements this year have cost nearly $6,000; and this, with the expenses of maintenance and interest on the mortgage, leaves the princely sum of $1,400 to be divided among the seven members of the permanent faculty, while the adjunct faculty, on whom now comes much of the burden of the teaching, gets no pay, but the consciousness of duty well done. For two years since coming to this building, four members of the faculty have turned in their entire dividends to the maintenance and furnishings accounts, besides contributing $1,200 apiece toward the building fund Whatever, then, there may have been in the past, this is the state of affairs today. Nor is the outlook very reassuring. The income is not likely to increase very much, but the demands are certain to be greater.

Now, we are not going to cry "poor," and ask you for money. That is not the object of getting you here; but we do feel that we have a right to ask for your sympathy and moral support. In what business will you find men giving their time and their money, sacrificing much of the best that is in them to the training up of rivals? Where, except in the learned professions, will you find men giving all of the best of their hard-earned experience to those who are soon to be their competitors, and this without proper return? Every student who graduates from this college, goes out without having paid, in any adequate way, for the time and trouble and learning which has been bestowed upon him. Thus every alumnus owes to the faculty a debt of gratitude, which he ought in some slight sense at least to try to repay.

I cannot let the occasion pass without paying a deserved tribute to the younger men, the adjunct professors and instructors, who labor on, year after year, in a generous rivalry, without any hope of justly earned reward. The reflex advantages of teaching are sufficient for awhile, but after a few years and a few repetitions of the subject, are largely lost. It is a wonder to me that so many are willing to continue on at this somewhat thankless task with such industry and faithfulness. Nothing but a strong sense of duty and responsibility can compel such work, and this, I am sure, is the reason for it. They deserve the honest thanks of all.

If this be true of the younger men, it is also true of the faculty as a whole. I can assure you that those to whom the management is entrusted by the council of the university have a very deep and enduring sense of their responsibility. They feel that they have the honor and good name of the institution in their hands, and that they stand between the students on the one side, and the public and the profession on the other, to see that those who are awarded degrees are properly fitted according to the standards of today. This is a weighty responsibility. It costs much thought, much discussion and a large share of time for its proper fulfilment. The management of such an institution, with its large number of students and its great corps of teachers, entails an immense amount of work. Fortunately, there are no dissensions in our number, and there is a strong desire to help, whenever possible, among the associate faculty.

Can we not, then, all of us, sinking what may be a proper personal rivalry, join together to make this medical college equal to the very best in the land? We have the men, we have the desire and the willingness; we lack only the means.

With this statement of facts, cannot we enlist the active coöperation of you Alumni in trying in every way to build up the institution to which you clearly owe your allegiance? This can only be done by endowments. We must have more money if we are to keep up to the requirements of the times—money, not for ourselves, but for those who make it their life-work to teach, and without whom we cannot possibly get along. The chairs of anatomy, physiology, chemistry, pathology, bacteriology, and perhaps some others, must be adequately endowed. New laboratories and new apparatus are greatly needed, and these with our present resources we are utterly unable to provide.

How can endowments be secured? Not from the medical profession. If they give their time and knowledge and experience, that is enough; others must give the money. But they will not do it unless the profession is united in their support of the college, are proud of it, take an interest in it, and use their influence for the promotion of its welfare. The power of a united alumni would be quickly felt, and we might, through them, secure adequate endowments, which would enable us to keep pace with our rivals.

The University of Buffalo has a fair name and stands in the front rank of the medical schools of the country. You need not feel ashamed to be enrolled among her alumni. Will you not help us to keep her ever in the same rank, and so strengthen our hands that we may be able to turn out men and women better and better fitted for the arduous struggles with disease and death ?

In regard to the methods of instruction now employed, I think I can safely say that we are not only fully up to the times, but are far in advance of most of the schools of the country. This school was one of the first to adopt the laboratory, the recitation, the conference and the demonstration methods. The old didactic lecture has been largely supplanted with the newer methods. By them the teacher is brought much closer to the pupil, the pupil's interest is greatly stimulated, and they are taught to think and reason for themselves.

But enough on this subject. One object of our gathering is to welcome our adopted children. According to a promise given, and given most cheerfully, by the faculty, at the time of the union of the medical departments of the Buffalo and the Niagara Universities, the alumni of the latter institution are to be counted by us as our own. In behalf of the faculty, I bid them welcome. We sincerely trust that the Alumni Association, over which we, of course, have no control, will likewise gladly welcome their new adopted brethren. I hope some action will be taken tonight, if it can be done properly, from which there will result a union of the two alumni associations, as there has been of the two faculties.

The Alumni of Niagara need not hesitate to enlist under our banner, for they can rest assured that they will receive courteous treatment and be received fully into the family. There are advantages in this. It has ever been the policy of the Buffalo School to do everything possible for its graduates, whenever its influence could be extended. By joining the Alumni Association, the same opportunities and privileges will be extended to the Niagara Alumni. They will be considered in the making of appointments, and in other ways, as opportunity offers. Fitness and merit only will be the lines to decide the choice.

We should be glad to grant honorary degrees to such of the Niagara graduates as might care for them, but, unfortunately, the laws of the state make this impossible. Still, it is proposed to take such steps as are necessary to legally adopt the alumni of Niagara University. The proper authorities will be consulted and everything done to further this end.

In conclusion, I ask the Niagara Alumni who cast in their lot with us to unite with the other alumni in trying to help us to keep the united school ever in the front and worthy of so numerous and distinguished a body of graduates.

I must beg pardon for having taken up so much of your time when there are so many good things awaiting you, but I could not let the occasion pass without trying to remove certain misapprehensions and trying to set certain crooked lines straight.

I thank you for your attention, and now beg permission to introduce to you Dr. Z. J. Lusk, of Warsaw, president of the Alumni Association of the Buffalo College.

Dr. Z. J. Lusk, '75, of Warsaw, N. Y., president of the alumni association of the University of Buffalo, responded in a few well chosen sentences. Dr. Mann then introduced Dr. W. G. Taylor, of Buffalo, president of the alumni association of Niagara University, who made a graceful and appropriate speech.

Upon motion of Dr. G. W. Wende, the association then went into executive session, whereupon he offered an amendment to the constitution, admitting the graduates of Niagara University to membership. The motion was received and the amendment will lie upon the table until the annual meeting in May. When this action is finally approved, which it no doubt will be at that time, the amalgamation of the two alumni associations will be accomplished. The informal session was then resumed.

A large number of letters of regret were received from all parts of the country from members of the classes from 1848 up to 1898. Dr. Kerr read extracts from some of the letters, which proved entertaining as showing the continued interest taken by the members in the institution.

The address of the evening was to have been delivered by Dr. Frederick Peterson, of New York, but owing to illness he was obliged to recall his promise, which he did in a most appropriate and witty letter of regret, and which Dr. Lytle read in full. In Dr. Peterson's stead Dr. William Warren Potter, of Buffalo, addressed the assembled audience for about twenty minutes, after which adjournment was had to the library, where an hour was spent at supper, during which the orchestra played many strains of excellent music. And so ended a most delightful evening. There were many expressions of satisfaction on all sides at the success of the reception, and it is hoped that each year may witness a repetition thereof on Washington's birthday. Among those who attended from out of town besides President Lusk were: Dr. T. D. Strong, '51, Westfield; Dr. M. A. Veeder, '83, Lyons; Dr. O. A. Tompkins, '65, Randolph, N. Y.; Dr. J. Wenz, '65, Lancaster, N. Y.; Dr. R. T. Rolph, '73, Dunkirk, N. Y.; Dr. Charles A. Ellis, '91, Sherman, N. Y.; Dr. William R. Campbell, '80, Niagara Falls, N. Y.; Dr.

Edward H. Ballou, '78, Gardenville, N. Y.; Dr. T. O. Burleson, '80, Bath, N. Y.; Dr. W. E. McChesney, '81, Pekin, N. Y.; Dr. James V. Otto, '78, Port Allegany, Pa.; Dr. James A. Taggart, '96, Salamanca, N. Y.; Dr. C. S. Payne, '84, Liberty, N. Y.; Dr. W. J. French, '84, Hamlet, N. Y.; Dr. J. B. Miller, '66, Alexander, N. Y.; Dr. C. A. Shepard, '96, Cherry Creek, N. Y.; Dr. M. G. Messenger, '94, Swormville, N. Y.; Dr. C. C. Mann, '95, Warsaw, N. Y.; Dr. B. E. Smith, '95, Angola, N. Y.; Dr. Mary E. Dickinson, '90, Rochester, N. Y.; Dr. William Stanton, '89, Varysburg, N. Y.; Dr. Mary J. Slaight, '80, Rochester, N. Y.; Dr. H. L. Clapp, '95, Geneva, N. Y.; Dr. W. B. Jolls, '95, Orchard Park, N. Y.; Dr. W. E. Palmer, '97, Le Roy, N. Y.; Dr. A. L. Mitchell, '98, East Aurora, N. Y.; Dr. Edith W. Stewart, '98, Hume, N. Y.; Dr. W. G. Austin, '95, Perry, N. Y.; Dr. L. L. Eddy, '97, Olean, N. Y.

The executive committee has addressed an appropriate note of thanks to each and every person who took part, or in any manner contributed to the success of the reunion.

